# Physiological and Immunomodulatory Effects of Purslane Extract in *Cirrhinus mrigala* Juveniles: Implications for Sustainable Production

**DOI:** 10.3390/ani15091334

**Published:** 2025-05-06

**Authors:** Muhammad Faisal, Syed Makhdoom Hussain, Shafaqat Ali, Dariusz Kucharczyk, Khalid A. Al-Ghanim

**Affiliations:** 1 Fish Nutrition Laboratory, Department of Zoology, Government College University Faisalabad, Faisalabad 38000, Pakistan; saroyafaisal13@gmail.com; 2 Department of Environmental Sciences, Government College University Faisalabad, Faisalabad 38000, Pakistan; shafaqataligill@gcuf.edu.pk; 3 Department of Biological Sciences and Technology, China Medical University, Taichung 40402, Taiwan; 4 Department of Research and Development, Chemprof, Gutkowo 54B, 11-041 Olsztyn, Poland; dariusz.kucharczyk@uwm.edu.pl; 5 Department of Ichthyology and Aquaculture, University of Warmia and Mazury in Olsztyn, Al. Warszawska117A, 10-957 Olsztyn, Poland; 6 Department of Zoology, College of Science, King Saud University, Riyadh 11451, Saudi Arabia; kghanim@ksu.edu.sa

**Keywords:** medicinal herb, extract, purslane, growth, enzyme activity, immunity

## Abstract

Medicinal plants are rich in nutrients and bioactive compounds, making them potentially suitable for use as chemotherapeutic agents and as additives in aquafeed. Our research evaluated the effects of purslane (*Portulaca oleracea*) extract supplemented in sunflower-based diets on the growth, carcass composition, blood indices, mineral content, liver antioxidant enzyme profile, and immune response in *Cirrhinus mrigala* (8.26 *±* 0.07 g/fish). The juveniles were fed one of seven different diets—T_0_ (no extract), T_1_ (0.5%), T_2_ (1%), T_3_ (1.5%), T_4_ (2%), T_5_ (2.5%), and T_6_ (3%)—twice daily, at a feeding rate of 5% of their live body weight. They were stocked in V-shaped steel tanks for 90 days, with 15 juveniles per tank and three replicates per dietary treatment. The results of this research revealed that 1–2% purslane extract substantially enhanced growth indices in fish (*p <* 0.05). Furthermore, the supplementation of 1–2% dietary purslane extract in the diet significantly lowered fat content and improved protein content (*p <* 0.05) compared to the diets with 0% and 3% purslane extract inclusion. The outcomes also indicated that the hematology and mineral content in the bodies of juveniles were significantly improved (*p <* 0.05) at all levels of purslane supplementation, relative to the control groups with 0% and 3% inclusion levels. Moreover, the administration of purslane extract markedly increased the liver antioxidant profile, including glutathione peroxidase, superoxide dismutase, and catalase. Additionally, there was a notable reduction in malondialdehyde levels when fish were fed diets having 1% and 1.5% extract. The findings of this study also revealed improvements in immunological markers, characterized by increased lysozyme activity and elevated total globulin levels. The current research suggests that supplementing *C. mrigala* diets with 1% purslane extract optimally enhances growth and immunity.

## Simple Summary

Medicinal plants provide both therapeutic and aquaculture benefits due to their nutrient-rich and bioactive profiles. This study describes the efficacy of purslane extract supplementation in enhancing the growth, carcass quality, hematological characteristics, mineral content, antioxidant enzymes status, and immune responses in *Cirrhinus mrigala*. Overall results showed that optimal growth, digestibility, blood profile, antioxidant activity, and immune responses were observed at supplementation of 1% purslane extract. In conclusion, based on our findings, 1% purslane supplementation can be recommended in *C. mrigala* diets, with no adverse effects on animal health and productivity.

## 1. Introduction

*Cirrhinus mrigala*, commonly called mrigal or nain, is a species of major carp that is extensively distributed in freshwater habitats across the sub-continent. As a bottom feeder, this species plays a vital role in polyculture [1]. Due to the worldwide scarcity of natural protein sources, fish has become an increasingly important food source. Notably, aquaculture accounts for around 50% of global fish production for human consumption [2]. Aquaculture, involving the rearing of aquatic animals, is crucial for meeting the increasing global demand for animal protein and enhancing food security [3]. However, the growth of this sector is accompanied by challenges such as optimizing fish growth rates and maintaining their health and welfare. The primary objective of sustainable and eco-friendly aquaculture is to minimize environmental damage while maximizing production and economic benefits [4,5].

Incorporating weeds in fish feed offers a viable option for sustainable aquaculture practices [6,7]. Due to increasing demand for nutritious feed and additives, there is an urgent need to explore novel methods for administering plant extracts rich in bioactive compounds [8]. Feed supplements play a crucial role in fish feed formulation because they are integral part of the fish diet, thereby enhancing the growth of farmed fish [9,10]. The dietary inclusion of plant-derived ingredients can enhance growth performance, survival rates, and immune function in farmed fish [11,12].

Herbs and their derivatives are valuable conventional medicines and dietary supplements that offer numerous benefits for animals, including growth promotion, immune stimulation, antibacterial activity, appetite stimulation, and stress reduction [13]. Purslane (*Portulaca oleracea*) is a highly adaptable succulent plant that thrives in diverse environments, from dry to damp conditions. It can be found growing on roadsides, in gardens, in orchards, and even on saline-alkaline soils [14,15]. Moreover, purslane has been found to exhibit over 30 diverse biological properties and has been utilized for more than 60 medicinal uses [16]. Previous research has indicated that purslane is rich in various phytochemicals and bioactive compounds, including alkaloids, antioxidants, minerals, phenolic compounds, ω-3 fatty acids, flavonoids, polysaccharides, and organic acids [17]. Purslane extract boasts a notable nutritional profile, featuring approximately 3.8% ash, 0.82% fiber, and 4.9% protein content. Additionally, it is rich in carotenoids, with a concentration of 40.40 mg per 100 g. The nutritional value of purslane is further highlighted by its dried powder form, which contains around 18.58% protein, 16.5% ash, and 17.9% fiber. The total carotenoid content in the dried powder, on a dry weight basis, is approximately 110.97 mg per 100 g [18].

Previous studies have demonstrated that dietary purslane supplementation enhances growth and health in fish. Several parts of the purslane plant, such as the roots and stems, have been found to possess medicinal properties [19,20]. Furthermore, the impact of purslane as a therapeutic herb on growth, antioxidant activity, and immune function has been investigated in gilthead seabream and Nile tilapia, with promising results in aquaculture [20,21].

Based on existing knowledge, this research is a pioneering investigation of the potential of purslane extract for *C. mrigala* juveniles, specifically examining its effects on growth, carcass composition, hematological indices, mineral content, antioxidant status, and immunological responses.

## 2. Materials and Methods

This present investigation was conducted at the Fish Nutrition Lab, GC University, Faisalabad, Punjab, Pakistan, over a period of 90 days.

### 2.1. Declaration of Ethics

The research obtained ethical approval from the Government College University, Faisalabad’s Ethics Review Committee (ERC), Faculty of Life Sciences (Ref No. GCUF/ERC/436), and was performed in compliance with ARRIVE guidelines.

### 2.2. Fish Rearing

The laboratory work was conducted at GC University Faisalabad, Pakistan. Fish (N = 315; average weight/ fish: 8.26 *±* 0.07 g) were procured from a nearby fish farm and transported to the experimental area. Thereafter, fish were relocated to trial tanks and acclimated to laboratory settings for 15 days. Prior to the feeding investigation, the juveniles were treated with a solution containing 5 g of sodium chloride per liter to eradicate parasites and prevent microbial infections [22]. At the time of acclimation, the juveniles were fed a basal diet continuously until they appeared satiated [23]. The trial tanks, each with a 70 L water capacity, were subjected to daily water exchanges and continuous aeration (24 h/day) via a capillary system. Moreover, water condition was assessed daily, with measurements of dissolved oxygen (7.5 ± 0.54 mg/L), temperature (27.5 ± 1.5 °C), and pH (7.7 ± 0.24) recorded.

### 2.3. Study Design

In this investigation, seven experimental diets were prepared, incorporating purslane as a key ingredient. The seven groups consisted of a control group (T_0_) fed a basal diet formulated with sunflower meal and six treatment groups (T_1_–T_6_) receiving diets supplemented with purslane extract at concentrations ranging from 0.5% to 3%. The treatment groups were comprised of three replicates, with 15 juveniles in each tank (45 fish/group), in a completely randomized design. The fish were fed sunflower-based diets with purslane extract supplementation twice daily at 5% of their body weight. Following the 2 h feeding session, excess feed residue was eliminated from the tanks via a valve-controlled drainage system, ensuring accurate assessment of feed intake and maintenance of optimal water quality parameters. Once the tanks were meticulously cleaned to remove leftover feed, they were filled with fresh water.

### 2.4. Purslane Extract Preparation

Purslane was collected from Kot Sultan, Punjab, Pakistan, and authenticated by the Botany Department at GCUF. The plant material was thoroughly washed to remove dirt, dust, and contaminants, and damaged parts were discarded. After drying, the material was powdered using an electric grinder and kept in a dry and airtight container [24].

The purslane extract was prepared via Soxhlet extractor or rotavap (J.P. Selecta, s.a; serial #. 0481090) using 2000 g of powdered purslane and a 60% ethanol solution (volume/volume ratio) as the solvent. Following extraction, a rotary evaporator (Scilogex RE 100-S) was used to concentrate the resulting mixture under reduced pressure, and the prepared extract was stored at −20 °C for later use [25].

### 2.5. Formulation of Diet Pellets

The diet ingredients were crushed, and then all components, including the extract, were mixed for five minutes. The mixture was then moistened with 10–15% water and blended with fish oil to achieve optimal dough consistency. The dough was then pelleted to form compact feed pellets [26]. The formulation and chemical assessment of test diets are illustrated in Table 1 and Table 2.

### 2.6. Proximate Analysis

The carcass composition of juveniles, including crude protein, moisture, crude fat, and ash content, was determined following standard protocols outlined by AOAC [27] and Yousaf et al. [28], using 1 g samples. Crude protein was assessed via micro Kjeldahl apparatus, with a nitrogen conversion factor of 6.25. Body samples were oven-dried at 105 °C for 12 h to evaluate moisture content. Crude fiber was analyzed through a digestion process with 1.25% NaOH and 1.25% H_2_SO_4_, followed by ignition of dried residues. Crude fat was determined using the Soxhlet apparatus with petroleum ether extraction technique. Gross energy content was evaluated using an adiabatic oxygen bomb calorimeter. Moreover, ash was determined by combusting samples in an electric furnace (650 °C) for 12 h (Eyela-TMF 3100).

### 2.7. Study of Growth

To calculate growth rates, fish from each tank were weighed at the commencement and end of the trial. The following standard formulae were utilized to measure fish growth indices such as feed conversion ratio (FCR), weight gain (WG), specific growth rate (SGR), and weight gain percentage (WG%), as calculated by Yousaf et al. [28]. Additionally, the following formulae from Faisal et al. [29] were applied to determine the survival rate, feed intake, and protein efficiency ratio (PER):

$$\mathrm{Feed}\;\mathrm{intake}\;(\frac{\frac{g}{\mathrm{fish}}}{\mathrm{day}})=\mathrm{total}\;\mathrm{feed}\;\mathrm{consumed}\;\mathrm{per}\;\mathrm{tank}/\mathrm{total}\;\mathrm{no}.\mathrm{of}\;\mathrm{fish}\;\mathrm{per}\;\mathrm{tank}\;\mathrm{PER}=\mathrm{gain}\;\mathrm{in}\;\mathrm{weight}\;(g)/\mathrm{protein}\;\mathrm{intake}\;\mathrm{in}\;\mathrm{feed}\;(g)\;\mathrm{Survival}\;\mathrm{rate}\;\mathrm{of}\;\mathrm{juveniles}\;(\%)=100\;(\mathrm{final}\;\mathrm{fish}\;\mathrm{number}/\mathrm{initial}\;\mathrm{fish}\;\mathrm{number})\;\mathrm{SGR}=(\mathrm{In}\;(\mathrm{final}\;\mathrm{weight},\mathrm{FW})-\mathrm{In}\;(\mathrm{initial}\;\mathrm{weight},\mathrm{IW}))\times 100/\mathrm{trial}\;\mathrm{period}\;\mathrm{FCR}=\mathrm{total}\;\mathrm{dry}\;\mathrm{feed}\;\mathrm{intake}\;(g)/\mathrm{wet}\;\mathrm{WG}\;\mathrm{WG}\;(\%)=(\mathrm{FW}-\mathrm{IW})\times 100/\mathrm{IW}\;\mathrm{WG}\;(g)=\mathrm{FW}-\mathrm{IW}$$

### 2.8. Hematological Studies

For each tank, three fish were randomly selected (nine fish per treatment group, with three replicates), and anesthesia was induced using clove oil (60 mg/L, 5 min exposure) [30]. Blood samples were drawn via caudal peduncle with sterile, heparinized needles. The micro-hematocrit method, as outlined by Brown [31], was employed to determine hematocrit or packed cell volume (PCV). A hemocytometer was employed to count white blood cells (WBCs), platelets (PLTs), and red blood cells (RBCs), as evaluated by Blaxhall and Daisley [32]. The procedures of Wedemeyer and Yastuk [33] were used to quantify the concentration of hemoglobin (Hb). Hematological indices such as mean corpuscular volume (MCV), MC hemoglobin (MCH), and MCH concentration (MCHC) were measured using these standard formulae as indicated by Hussain et al. [4].

### 2.9. Mineral Estimation

The mineral content of fish bodies was analyzed by oven-drying randomly collected fish samples from each tank, from which 1 g was taken (3 g per group). Sample preparation involved acid digestion using a 2:1 mixture of nitric and perchloric acids, without prior dilution, following AOAC guidelines [27]. The digested samples were filtered into 25 mL volumetric flasks, subsequently diluted to the mark with ultrapure water, and analyzed using atomic absorption spectroscopy.

### 2.10. Activity of Antioxidant Enzymes and Immune Response

Liver tissue samples from each group (n = 6) were processed through homogenization in Tris buffer (0.4 M, pH 7.0), followed by centrifugation (9400× *g*, 10 min), and the resulting supernatant was preserved at −20 °C for subsequent analysis. To measure SOD enzyme (superoxide dismutase) activity, a method based on inhibiting nitroblue-tetrazolium reduction was used, as described previously by Winterbourn et al. [34]. CAT (catalase) activity was evaluated by assessing its ability to break down hydrogen peroxide into water, as indicated by Claiborne [35]. GPx (glutathione peroxidase) activity was determined by monitoring glutathione oxidation and the corresponding decrease in absorbance, as suggested by Rotruck et al. [36]. Lipid peroxidation was assessed by detecting malondialdehyde content using a chromophore-based method [37].

Finally, for lysozyme (LYZ) and globulin (GLO) analysis, serum was collected from the same hematology samples (nine fish per treatment group) after centrifugation at 6000× *g* for 10 min and kept at −20 °C for further analysis. LYZ levels in serum were evaluated using an established technique by Ellis [38]. GLO levels in the serum were estimated with a Hitachi 7600-110 biochem analyzer.

### 2.11. Statistical Analyses

A microcomputer was used for performing statistical analysis on the growth responses, carcass, hematology, mineralization, liver antioxidant profile, and immune parameters of fish. The results were evaluated via one-Way ANOVA as described by Steel et al. [39]. Bartlett’s test was used to warrant the homogeneity of variance across groups prior to conducting ANOVA. Tukey’s HSD test was used for post-hoc comparisons, with *p <* 0.05 considered statistically significant [40]. The results are presented as means ± standard deviation (SD) to illustrate the precision of the estimated means.

## 3. Results

### 3.1. Assessment of Growth Performance and Survival

Table 3 outlines the efficacy of various doses of purslane extract on the growth of *C. mrigala*. The indices such as final weight, WG, WG%, feed intake, FCR, PER, survival rate and SGR were substantially improved. The dietary inclusion of 1–1.5% purslane extract substantially enhanced growth indices (*p <* 0.05). The current study also indicated that incorporating dietary purslane extract at 2.5%, 0%, and 3% significantly lowered growth performance. Moreover, juveniles fed diets containing 1–1.5% purslane extract exhibited notably better survival rates in contrast to those fed with 0%, 2.5%, or 3% purslane extract.

### 3.2. Whole-Body Nutrient Analysis

Table 4 shows the findings of whole body proximate analysis in fish. The outcomes revealed that the proximate analysis of fish was substantially enhanced (*p <* 0.05) in response to variations in the level of purslane extract in their diet. As the purslane extract level elevated (from 1% to 1.5%), there was a tendency of significant increased protein content, accompanied by decreased fat content compared to all other treatment groups. Alternatively, ash content improved slightly in the treatment groups receiving 1% to 1.5% purslane extract, and moisture levels varied across the different test diets.

### 3.3. Hematology

The findings of blood profile are illustrated in Table 5. With an increase in purslane extract from 1% to 1.5% in the diet of *C. mrigala*, the RBC count (3.86 ± 0.10 × 10^6^ mm^3^) and WBC count (8.06 ± 0.10 × 10^6^ mm^3^) were observed to be highest at the 1% extract concentration. The corresponding Hb and PCV values were 8.75 ± 0.10 g/100 mL and 29.44 ± 0.34%, respectively. The MCHC remained relatively constant across lower concentrations but decreased slightly at the 0% and 3% concentrations. In contrast, MCV peaked at 148.60 ± 6.94 fl at 3% extract concentration, while the MCH values varied across groups.

### 3.4. Whole-Body Mineralization

Dietary supplementation with purslane extract significantly enhanced whole-body mineral content in *C. mrigala* (*p* < 0.05) (Table 6). The findings reveal that the highest levels of minerals (Ca, P, Mg, Zn, K, Na, Mn, Fe, and Cu were recorded at 1% purslane extract, compared to the control and 3% levels. In contrast, the minimum values of body minerals were noted at 0% and 3% purslane extract concentrations, respectively.

### 3.5. Antioxidant Status

**Figure 1:**
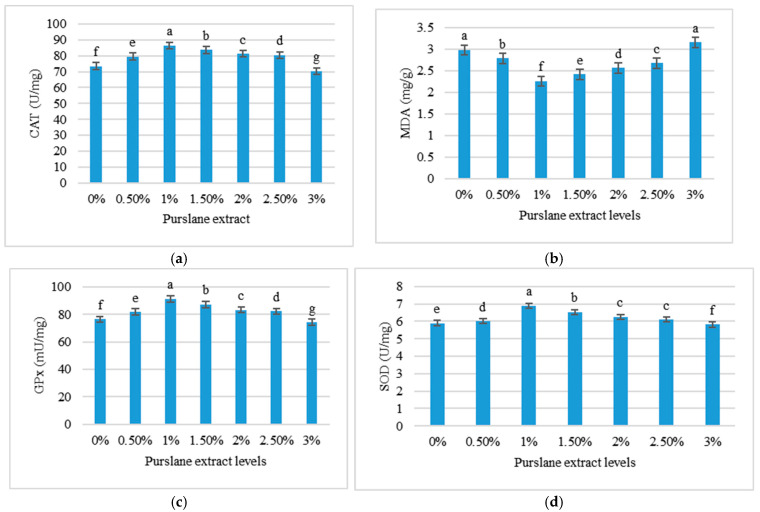
Liver antioxidant enzymes of *Cirrhinus mrigala* fed different concentrations of purslane extract: (**a**) CAT—catalase (U/mg); (**b**) MDA—malondialdehyde (mg/g); (**c**) GPx—glutathione peroxidase (mU/mg); and (**d**) SOD—superoxide dismutase (U/mg). Values are displayed as mean ± SD of triplicates. Distinct superscript letters (a–g) denote significant variations at *p* < 0.05.

Figure 1 highlights the impacts of purslane extract concentrations on liver antioxidant indices in *C. mrigala* after a three-month period. Notably, treatment T_2_ (1% purslane extract) exhibited the greatest CAT activity (86.41 ± 0.36 U/mg), SOD activity (6.90 ± 0.04 U/mg), and GPx activity (91.34 ± 0.14 mU/mg), as well as the lowest MDA level (2.26 ± 0.07 mg/g). In contrast, T_6_ treatment (3% purslane extract) showed the lowest antioxidant enzyme activity and the highest MDA levels, suggesting potential adverse effects at higher concentrations.

### 3.6. Immune Response

**Figure 2:**
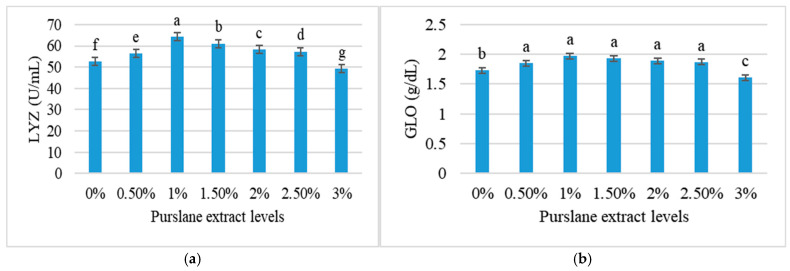
Immune responses of *Cirrhinus mrigala* fed different concentrations of purslane extract: (**a**) LYZ—lysozyme (U/mL) and (**b**) GLO—globulin (g/dL). Values are displayed as mean ± SD of triplicates. Distinct superscript letters (a–g) denote significant variations at *p* < 0.05.

Figure 2 represents the immunological response of *C. mrigala* to diets containing varied amounts of purslane extract over a three-month period. The 1% extract (T_2_) exhibited the highest LYZ activity at 64.31 ± 0.19 U/mL, with a GLO of 1.97 ± 0.06 g/dL. At higher concentrations, LYZ and GLO levels decreased, with 3% extract (T_6_) showing the lowest activity levels of 49.28 ± 0.16 U/mL for LYZ and 1.61 ± 0.08 g/dL for GLO, respectively.

## 4. Discussion

In intensive aquaculture systems, feeding techniques significantly impact production [41]. Therefore, using functional feed supplements could be a sustainable way to promote the well-being and productivity of farmed fish [10,11,42]. Therapeutic plants used as functional feed additives have resulted in various benefits for aquatic animals, including enhanced anti-oxidative activity, immunological response, and growth rate [43,44,45]. Purslane’s rich composition of bioactive compounds makes it a valuable dietary supplement for enhancing growth performance, metabolic function, and disease resistance in animals [46]. This pioneering research explores the efficacy of purslane extract on growth indices, nutrient utilization, blood profile, antioxidant status, and immune parameters in *C. mrigala* juveniles.

According to Sahin et al. [47], dietary supplementation with 0.9% whole plant purslane extract significantly improved growth and survival in goldfish. Furthermore, a study by Mohammadalikahni et al. [48] found that incorporating varying concentrations of dried purslane extract (1%) into the diet of rainbow trout improved their growth efficiency. Purslane’s nutrient profile, comprising macronutrients such as proteins, carbohydrates, lipids, and ash, along with bioactive compounds like carotenoids, flavonoids, lignins, and phenolic acids, contributes to its growth-promoting effects [49]. Likewise, Díaz-Vázquez et al. [50] found that dietary purslane supplementation significantly impacted Nile tilapia growth, with notable differences observed at 10% compared to the control. Our outcomes align with Ahmadifar et al. [51], who found that supplementing grass carp feed with 0.5% purslane leaves significantly improved growth performance. Purslane’s nutrient content, particularly its rich omega-3 fatty acid content, substantially enhances growth indices. Additionally, purslane is rich in vitamins C and E, as well as various B vitamins, which are essential for various metabolic processes [52]. Consistent with our findings, Faisal et al. [29] demonstrated that supplementing sunflower-based diets with *P. oleracea* whole-plant extract (1.5%) enhanced the growth performance in *L. rohita* fingerlings. Research by Wang et al. [46] showed that incorporating purslane into the feed of broilers had a positive impact on their growth performance. However, contradictory results were observed, as a 3% purslane diet significantly impaired growth and feed intake in Nile tilapia relative to the control [50]. The reduced consumption of purslane-supplemented feed is likely attributed to the decreased palatability of the purslane powder, which is characterized by its acidic pH profile. The elevated concentrations of organic acids in purslane, including oxalic, citric, and malic acids, may contribute to its unappealing taste to the fish. Furthermore, the presence of phytic acid in purslane can form insoluble complexes with essential micronutrients, including minerals, vitamins, and amino acids, within the gastrointestinal tract of the fish. This phytate-mineral binding may impair nutrient digestion, absorption, and utilization, ultimately leading to decreased feed intake and suboptimal growth in the fish [53]. Moreover, the inconsistencies in growth results could be attributed to variations in factors such as feeding duration, supplement dosage, and fish species.

According to Díaz-Vázquez et al. [50], supplementing Nile tilapia with purslane flour (10%) significantly enhanced their body composition. Incorporating purslane into fish feed at varying levels led to elevated protein and decreased fat content compared to the control. Similarly, the present research indicates that incorporating purslane extract into diets positively influenced the body composition of *C. mrigala* juveniles. Likewise, research by Faisal et al. [29] found that adding purslane whole-plant extract (1.5%) to SFM-based diets improvsed the body composition in *L. rohita* fingerlings. This improvement may be attributed to the bioactive components present in purslane, including fatty acids, vitamins, terpenoids, flavonoids, organic acids, minerals, and alkaloids [54]. Additionally, the high fiber content in purslane may also contribute to the enhanced body composition, as dietary fiber can help regulate digestive health, promote satiety, and improve nutrient utilization in fish [49].

The current study’s findings indicate that purslane extract supplementation in diets substantially improved the blood profile of *C. mrigala*. These outcomes align with previous studies, which have described the hematological benefits of purslane supplementation in various species. For instance, Mohammadlikhani et al. [48] exhibited significant enhancements in hematological parameters, including RBC, WBC, and Hb levels, in rainbow trout fed dietary purslane (1% dried extract). Our outcomes are supported by Faisal et al. [29], who demonstrated that whole-plant purslane extract (1.5) supplementation in sunflower-based diets positively impacted the hematological parameters in *L. rohita*. Similarly, Habibian et al. [55] reported that incorporating purslane into the feed of broiler chickens resulted in increased Hb concentration and RBC counts. Purslane is a rich source of iron, copper, and zinc, which are essential for hematopoiesis and maintaining healthy hematological parameters. Its immunomodulatory and anti-inflammatory properties regulate the immune system, promoting a balanced WBC and RBC ratio, while reducing inflammation. Additionally, purslane’s antioxidants (vitamins C and E, beta-carotene) protect erythrocytes from oxidative damage, enhancing RBC counts and Hb levels [48,49].

The incorporation of purslane extract in the feed of *C. mrigala* enhanced the overall mineral content of the fish’s body, likely due to its richness in n-3 fatty acids, phenolic acids, vitamins C and E, flavonoids, and minerals such as Mg, P, Na, K, and Ca [56,57]. Consistent with our research, Faisal et al. [29] showed that incorporating purslane whole-plant extract (1.5%) into sunflower-based diets led to improved mineralization in *L. rohita* fingerlings. However, Shalaei et al. [58] reported that purslane seed supplementation in hen diets did not substantially alter blood plasma levels of calcium, iron, phosphorus, or magnesium. In our study, the observed improvements in mineral composition associated with purslane supplementation warrant further investigation, particularly in addressing two key concerns: the potential inefficiency of the 80% Et-OH extraction method in releasing minerals from purslane, and the possibility of fish meeting their calcium requirements through environmental water, thereby casting doubt on the significance of purslane as a calcium source [59]. Future investigations could explore purslane’s role in modulating mineral composition, identify the key bioactive compounds responsible for its effects, and investigate the underlying mechanisms driving its benefits.

The increasing global interest in utilizing medicinal plants as immunostimulants and antioxidants in aquaculture parallels their remarkable biological active properties [60]. In this context, our study demonstrates that purslane enhances antioxidant enzyme activity (GPx, SOD, and CAT) in *C. mrigala*. These outcomes align with those of Ahmadifar et al. [51], who stated that supplementing grass carp fingerlings’ diets with 0.5% purslane seed extract boosted antioxidant activity and immunological response. Furthermore, Abdel-Razek et al. [21] observed that purslane leaf extract supplementation after *Aeromonas hydrophila* infection in Nile tilapia activated immune responses, leading to increased survival rates. Feeding Nile tilapia diets enriched with purslane enhances their antioxidant defenses. This is likely because purslane contains bioactive compounds like phenolic acids, alkaloids, and flavonoids, which are known to have strong antioxidant effects. Additionally, the immune-enhancing effects of purslane may be attributed to its rich mineral profile, ω-3 fatty acids, vitamins A and C, α-tocopherol, glutathione, and β-carotene [21,57].

Lysozyme activity is an essential component of the nonspecific immune defense system. Our study found that serum immunological indicators, including total immunoglobulin levels and lysozyme activity, increased significantly in *C. mrigala* fed diets supplemented with 1% purslane. These findings are aligned with Abdel-Razek et al. [21], who demonstrated that adding purslane leaf powder to the diet enhances lysozyme activity in Nile tilapia. Lysozyme is a vital bactericidal enzyme that plays essential role in the innate immune response of fish. This cationic enzyme breaks down the peptidoglycan layers of bacterial cell walls by specifically targeting and splitting the bonds between N-acetylmuramic acid and N-acetylglucosamine, resulting in the destruction of the bacterial cell [61]. Lysozyme levels increase in response to infections caused by various microorganisms, exhibiting not only bacteriolytic activity but also activating phagocytes and the complement system and, functioning as an opsonin to enhance pathogen elimination [21]. Dietary supplementation with purslane extract has been revealed to enhance immunological responses in various fish species. For instance, Ahmadifar et al. [51] identified that supplementing diets with 0.5% purslane extract notably enhanced immunological response in grass carp fingerlings. Similarly, Mohammadalikhani et al. [48] observed a substantial boost in immune function in rainbow trout fry fed a diet containing 1.5% purslane extract.

## 5. Conclusions

This study demonstrates that adding 1–2% purslane extract supplementation significantly improves growth, carcass composition, hematological parameters, body mineralization, hepatic enzyme activities, and immune responses in *C. mrigala*. Notably, the present study reveals that a 1% purslane-extract-supplemented diet is optimal for enhancing growth and immunity in *C. mrigala*, thereby supporting sustainable aquaculture practices. Future research should elucidate the molecular mechanisms underlying purslane extract’s impact on *C. mrigala* performance, including modulation of gene expression, hormone regulation, and alterations in metabolic pathways.

## Figures and Tables

**Table 1:** Experimental feed and ingredient composition (%) incorporating purslane extract at different doses (dry matter basis).

Feed components							
(%)	T0 (Control)	T1	T2	T3	T4	T5	T6
Sunflower meal	54	54	54	54	54	54	54
Rice polish	8	8	8	8	8	8	8
Vitamin Premix *	1	1	1	1	1	1	1
Fish meal	17	17	17	17	17	17	17
Fish oil	6	6	6	6	6	6	6
Chromic oxide	1	1	1	1	1	1	1
Ascorbic acid	1	1	1	1	1	1	1
Mineral mixture **	1	1	1	1	1	1	1
Wheat flour ***	11	10.5	10	9.5	9	8.5	8
Purslane extract	0	0.5	1	1.5	2	2.5	3
Proximate Composition of diets							
Crude protein (%)	30.34	30.43	30.37	30.31	30.38	30.35	30.39
Crude lipid (%)	8.06	8.11	8.07	8.08	8.06	8.08	8.11
Gross energy (Kcal/g)	3.43	3.46	3.42	3.45	3.47	3.43	3.44
Proximate Composition of ingredients							
	Gross energy (Kcal/g)	Crude protein (%)	Crude fat (%)	Dry matter (%)	Carbohydrates (%)	Crude fiber (%)	Ash (%)
Sunflower meal	3.43	43.53	10.50	91.51	25.78	10.65	9.54
Wheat flour	3.48	10.76	2.10	92.39	82.87	2.12	2.15
Rice, Polish	3.53	11.24	13.63	92.27	50.18	13.19	11.76
Fish meal	3.31	52.67	6.35	91.43	19.43	1.38	20.17

* vitamin (Vit.) premix∙kg^−1^: vit. D3: 3,000,000 IU, folic acid: 1300 mg, nicotinic acid: 59,000 mg, A: 16,000,000 IU, B6: 3900 mg, B2: 4000 mg, Ca pantothenate: 13,000 mg, B12: 40 mg, B1: 3500 mg, K: 1200 mg, E: 30,000 mg, choline: 35,000,000 µg, biotin: 300,000 µg. ** Mineral premix∙kg^−1^: Fe: 1100 mg, P: 136 g, Ca: 156 g, Mg: 56 g, Se: 2.5 mg, Mn: 2000 mg, Na: 44 g, Zn: 3000 mg, Co: 42 mg, Cu: 600 mg, I: 39 mg. *** Purslane supplement was included at the expense of wheat flour.

**Table 2:** Chemical composition of purslane extract: proximate (%) and mineral (ppm) analysis.

Nutrient content (%)	
Moisture	12.34
Crude fat	4.27
Carbohydrates	42.51
Ash	2.13
Crude protein	18.52
Crude fiber	20.64
Mineral content (ppm)	
Iron	22
Phosphorus	330
Potassium	650
Calcium	661
Magnesium	657

**Table 3:** The growth responses of *Cirrhinus mrigala* in response to varying doses of dietary purslane extract.

Purslane Extract	Treatments	Initial Weight (g)	Final Weight (g)	Weight Gain (WG, g)	FCR	SGR	Feed Intake (g/Fish/Day)	WG (%)	PER	Survival Rate (%)
0%	T_0_ (Control)	8.37 ± 0.09 ^a^	18.50 ± 0.30 ^f^	10.13 ± 0.35 ^e^	2.11 ± 0.17 ^ab^	0.88 ± 0.03 ^e^	0.24 ± 0.01 ^d^	121.14 ± 5.03 ^e^	0.32 ± 0.01 ^f^	96
0.5%	T_1_	8.34 ± 0.08 ^a^	19.32 ± 0.16 ^e^	10.98 ± 0.13 ^e^	1.88 ± 0.03 ^bc^	0.93 ± 0.01 ^e^	0.23 ± 0.00 ^d^	131.66 ± 2.01 ^e^	0.36 ± 0.01 ^e^	100
1%	T_2_	8.23 ± 0.05 ^ab^	29.54 ± 0.33 ^a^	21.31 ± 0.30 ^a^	1.32 ± 0.05 ^d^	1.42 ± 0.01 ^a^	0.31 ± 0.02 ^a^	258.82 ± 2.80 ^a^	0.70 ± 0.00 ^a^	100
1.5%	T_3_	8.32 ± 0.05 ^ab^	27.08 ± 0.23 ^b^	18.77 ± 0.21 ^b^	1.41 ± 0.06 ^d^	1.31 ± 0.01 ^b^	0.29 ± 0.02 ^ab^	225.65 ± 2.34 ^b^	0.61 ± 0.01 ^b^	100
2%	T_4_	8.21 ± 0.08 ^ab^	23.79 ± 0.20 ^c^	15.58 ± 0.28 ^c^	1.58 ± 0.11 ^cd^	1.18 ± 0.02 ^c^	0.27 ± 0.02 ^bc^	189.68 ± 5.10 ^c^	0.51 ± 0.01 ^c^	99
2.5%	T_5_	8.12 ± 0.09 ^b^	20.52 ± 0.31 ^d^	12.39 ± 0.37 ^d^	1.74 ± 0.08 ^c^	1.03 ± 0.03 ^d^	0.24 ± 0.01 ^cd^	152.61 ± 6.10 ^d^	0.41 ± 0.01 ^d^	99
3%	T_6_	8.29 ± 0.09 ^ab^	16.69 ± 0.38 ^g^	8.40 ± 0.45 ^f^	2.43 ± 0.20 ^a^	0.78 ± 0.04 ^f^	0.23 ± 0.02 ^d^	101.33 ± 6.36 ^f^	0.27 ± 0.01 ^g^	95

Values are displayed as mean ± SD of triplicates. Columns with distinct letters denote significant variations at *p* < 0.05.

**Table 4:** Proximate composition of *Cirrhinus mrigala* in response to varying doses of dietary purslane extract.

Purslane Extract	Treatments	Protein (%)	Ash (%)	Fat (%)	Moisture (%)
0%	T_0_ (Control)	13.28 ± 0.14 ^d^	1.83 ± 0.09 ^d^	5.43 ± 0.16 ^ab^	78.88 ± 0.10 ^a^
0.5%	T_1_	13.52 ± 0.17 ^d^	2.50 ± 0.07 ^ab^	5.36 ± 0.13 ^ab^	78.46 ± 0.13 ^ab^
1%	T_2_	15.37 ± 0.16 ^a^	2.63 ± 0.14 ^a^	3.20 ± 0.22 ^e^	78.34 ± 0.10 ^ab^
1.5%	T_3_	15.06 ± 0.18 ^ab^	2.62 ± 0.10 ^a^	3.83 ± 0.11 ^d^	77.89 ± 0.12 ^c^
2%	T_4_	14.63 ± 0.12 ^bc^	2.36 ± 0.06 ^b^	4.27 ± 0.11 ^c^	78.21 ± 0.20 ^b^
2.5%	T_5_	14.30 ± 0.20 ^c^	2.28 ± 0.08 ^bc^	5.09 ± 0.18 ^b^	78.20 ± 0.15 ^b^
3%	T_6_	13.07 ± 0.21 ^d^	2.05 ± 0.09 ^cd^	5.63 ± 0.16 ^a^	78.84 ± 0.17 ^a^

Values are displayed as mean ± SD of triplicates. Columns with distinct letters denote significant variations at *p* < 0.05.

**Table 5:** The hematology of *Cirrhinus mrigala* in response to varying doses of dietary purslane extract.

Purslane Extract	Treatments	RBCs (10^−6^ mm^−3^)	Hb (g/100mL)	PCV (%)	WBCs (10^−6^ mm^−3^)	MCV (fl)	MCH (pg)	PLT	MCHC (%)
0%	T_0_ (Control)	1.63 ± 0.12 ^e^	6.95 ± 0.09 ^e^	21.57 ± 0.25 ^f^	6.63 ± 0.0.09 ^de^	133.02 ± 8.58 ^b^	42.89 ± 0.3.81 ^a^	56.39 ± 0.0.06 ^f^	32.21 ± 0.76 ^a^
0.5%	T_1_	2.09 ± 0.08 ^d^	7.42 ± 0.10 ^d^	22.43 ± 0.17 ^e^	6.90 ± 0.06 ^cd^	107.23 ± 3.06 ^c^	35.48 ± 0.79 ^b^	58.62. ± 0.13 ^e^	33.08 ± 0.24 ^a^
1%	T_2_	3.86 ± 0.10 ^a^	8.75 ± 0.10 ^a^	29.44 ± 0.34 ^a^	8.06 ± 0.10 ^a^	76.34 ± 1.25 ^e^	22.71 ± 0.80 ^e^	66.86 ± 0.23 ^a^	31.81 ± 3.43 ^a^
1.5%	T_3_	3.10 ± 0.14 ^b^	8.30 ± 0.08 ^b^	26.82 ± 0.28 ^b^	7.74 ± 0.15 ^b^	86.59 ± 3.29 ^de^	26.79 ± 0.93 ^de^	64.74 ± 0.06 ^b^	30.93 ± 0.21 ^a^
2%	T_4_	2.77 ± 0.07 ^c^	7.89 ± 0.14 ^c^	24.08 ± 0.18 ^c^	7.46 ± 0.12 ^b^	86.95 ± 1.85 ^de^	28.50 ± 1.20 ^cd^	62.77 ± 0.11 ^c^	32.77 ± 0.68 ^a^
2.5%	T_5_	2.33 ± 0.04 ^d^	7.59 ± 0.06 ^d^	23.10 ± 0.21 ^d^	7.09 ± 0.08 ^c^	99.17 ± 2.57 ^cd^	32.56 ± 0.33 ^bc^	59.37 ± 0.07 ^d^	32.84 ± 0.52 ^a^
3%	T_6_	1.52 ± 0.08 ^e^	6.75 ± 0.09 ^e^	22.50 ± 0.15 ^de^	6.52 ± 0.10 ^e^	148.60 ± 6.94 ^a^	44.59 ± 2.75 ^a^	55.57 ± 0.11 ^g^	29.99 ± 0.60 ^a^

RBC = red blood cell, Hb = hemoglobin, PCV = packed cell volume, WBC = white blood cell, MCV = mean corpuscular volume, MCH = mean corpuscular hemoglobin, PLT = platelet, MCHC = mean corpuscular hemoglobin concentration. Values are displayed as mean ± SD of triplicates. Columns with distinct superscript letters denote significant variations at *p <* 0.05.

**Table 6:** The body mineral status of *Cirrhinus mrigala* in response to varying doses of dietary purslane extract.

Purslane Extract	Treatments	Ca (%)	Mg (%)	Na (mg/g)	Cu (μg/g)	K (%)	Fe (μg/g)	P (%)	Mn (μg/g)	Zn (μg/g)
0%	T_0_ (Control)	0.68 ± 0.15 ^bc^	2.78 ± 0.14 ^bc^	4.91 ± 0.13 ^de^	3.06 ± 0.11 ^d^	4.79 ± 0.07 ^de^	43.49 ± 0.15 ^f^	0.78 ± 0.16 ^bc^	4.89 ± 0.10 ^ef^	2.99 ± 0.07 ^de^
0.5%	T_1_	0.74 ± 0.12 ^abc^	2.89 ± 0.21 ^abc^	5.07 ± 0.10 ^cde^	3.21 ± 0.05 ^cd^	4.98 ± 0.16 ^de^	45.38 ± 0.13 ^e^	0.82 ± 0.14 ^bc^	5.23 ± 0.12 ^e^	3.13 ± 0.14 ^cde^
1%	T_2_	1.06 ± 0.10 ^a^	3.35 ± 0.15 ^a^	5.78 ± 0.15 ^a^	4.01 ± 0.16 ^a^	7.85 ± 0.10 ^a^	61.59 ± 0.15 ^a^	1.26 ± 0.16 ^a^	10.71 ± 0.23 ^a^	3.99 ± 0.14 ^a^
1.5%	T_3_	0.94 ± 0.10 ^ab^	3.21 ± 0.17 ^ab^	5.62 ± 0.15 ^ab^	3.74 ± 0.09 ^ab^	6.18 ± 0.11^b^	56.62 ± 0.17 ^b^	1.10 ± 0.08 ^ab^	8.93 ± 0.14 ^b^	3.70 ± 0.15 ^ab^
2%	T_4_	0.86 ± 0.10 ^abc^	3.09 ± 0.14 ^abc^	5.46 ± 0.11 ^abc^	3.50 ± 0.12 ^bc^	5.46 ± 0.13 ^c^	52.01 ± 0.11 ^c^	0.98 ± 0.15 ^abc^	6.91 ± 0.18 ^c^	3.45 ± 0.14 ^bc^
2.5%	T_5_	0.78 ± 0.13 ^abc^	2.98 ± 0.17 ^abc^	5.27 ± 0.26 ^bcd^	3.36 ± 0.09 ^c^	5.12 ± 0.21 ^cd^	48.38 ± 0.16 ^d^	0.88 ± 0.06 ^abc^	5.76 ± 0.09 ^d^	3.26 ± 0.11 ^cd^
3%	T_6_	0.60 ± 0.12 ^c^	2.67 ± 0.16 ^c^	4.73 ± 0.13 ^e^	2.75 ± 0.09 ^e^	4.65 ± 0.12 ^e^	39.34 ± 0.11 ^g^	0.62 ± 0.19 ^c^	4.71 ± 0.12 ^f^	2.81 ± 0.09 ^e^

Values are displayed as mean ± SD of triplicates. Columns with distinct superscript letters denote significant variations at *p* < 0.05.

## Data Availability

The data that support this study will be available from the corresponding author upon reasonable request.
